# Supplementary material for: Quantum squeezing amplification with a weak Kerr nonlinear oscillator
Source: Nat Commun. 2025 Dec 18;17:970. doi: 10.1038/s41467-025-67699-0 (PMC12848120; doi:10.1038/s41467-025-67699-0)
Supplement: Supplementary file 1 — Supplementary Information [file 41467_2025_67699_MOESM1_ESM.pdf]

Supplementary Information for "Quantum squeezing amplification with a weak Kerr nonlinear oscillator"

Yanyan Cai,[1,2,*] Xiaowei Deng,[2,*] Libo Zhang,[1,2,*] Zhongchu Ni,[2] Jiasheng Mai,[1,2] Peihao Huang,[2] Pan Zheng,[2] Ling Hu,[2,3] Song Liu,[2,3] Yuan Xu,[2,3,†] and Dapeng Yu[2,3]

[1]*Shenzhen Institute for Quantum Science and Engineering, Southern University of Science and Technology, Shenzhen 518055, China*
[2]*International Quantum Academy, Shenzhen 518048, China*
[3]*Shenzhen Branch, Hefei National Laboratory, Shenzhen 518048, China*

Supplementary Note 1 - Experimental device and setup

We experimentally demonstrate quantum squeezing amplification in a three-dimensional (3D) circuit quantum electrodynamics (QED) architecture [1]. The experimental device is similar to that in Refs. [2, 3] and comprises three key components: a 3D coaxial stub cavity [4], a fixed-frequency superconducting transmon qubit [5], and a Purcell-filtered stripline readout resonator [6], as illustrated in Supplementary Figure 1.

The 3D circuit QED device is directly machined from a high-purity (5N5) aluminum block and undergoes chemical etching to extend the cavity's coherence lifetime [7]. The coaxial stub cavity (C) is designed as a 3D $\lambda/4$ transmission line resonator, whose fundamental mode is utilized as the storage cavity for storing the generated squeezed states. The superconducting transmon qubit (Q), fabricated on a sapphire chip with two antenna pads, serves as an auxiliary qubit. One pad couples to the storage cavity, while the other couples to the stripline readout resonator (R). The coupling to the nonlinear qubit induces a weak Kerr nonlinearity to the storage cavity, effectively realizing a Kerr nonlinear oscillator. The Purcell-filtered readout resonator consists of two planar $\lambda/2$ striplines: one is strongly coupled to the transmon qubit for fast dispersive readout of qubit states, and the other is coupled to the external environment, acting as a Purcell filter to preserve the coherence lifetimes of both the qubit and the storage cavity.

The experimental device is placed inside a magnetic shield in a cryogen-free dilution refrigerator at a temperature below 10 mK. The control wiring of the system is similar to that in Ref. [8]. All microwave control pulses for the auxiliary qubit, storage cavity, and readout resonator are generated using single-sideband in-phase and quadrature (IQ) modulations with an arbitrary waveform generator (Tektronix AWG5208). These signals are transmitted to the experimental device through coaxial cables with microwave isolators, filters, and attenuators to minimize reflection waves and radiation noise. The transmitted readout signal is amplified by a high electron mobility transistor (HEMT) amplifier at 4K stage, followed by a standard commercial radio frequency (RF) amplifier at room temperature. Subsequently, the signal is downconverted using a microwave mixer with the same local oscillator (LO) frequency as that used to generate the readout pulse and is recorded and digitized by a data acquisition card (AlazarTech ATS9870) along with corresponding reference signals. Detail information on the wiring and experimental setup is provided in Supplementary Figure 1.

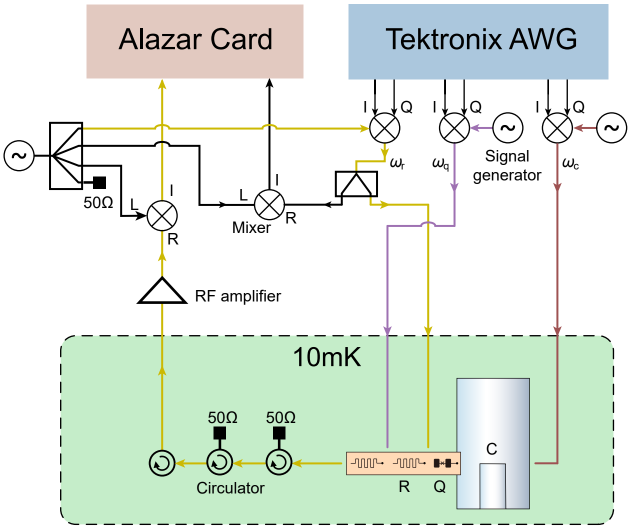

Supplementary Figure 1: Control wiring and experimental setup.

Supplementary Note 2 - Quantum dynamics of the detuned driven Kerr oscillator

A. System Hamiltonian and parameters

In this 3D circuit QED system, the superconducting qubit is dispersively coupled to both the storage cavity and the readout resonator, with the dispersive Hamilto-

*These authors contributed equally to this work.
†Electronic address: xuyuan@iqasz.cn

Supplementary Table 1: Device parameters of the system.

Parameters	Value
Storage cavity frequency $\omega_{\rm c}/2\pi$(GHz)	6.60
Storage cavity Kerr nonlinearity $K/2\pi$(kHz)	5.83
Storage cavity dispersive shift $\chi_{\rm qc}/2\pi$(MHz)	1.94
Storage cavity relaxation $T_{1,\rm c}$ (μs)	395
Storage cavity Ramsey coherence $T_{2,\rm c}$ (μs)	595
Storage cavity thermal population	0.1%
Qubit frequency $\omega_{\rm q}/2\pi$(GHz)	5.28
Qubit anharmonicity $\eta_{\rm q}/2\pi$(MHz)	194.6
Qubit relaxation $T_{1,\rm q}$ (μs)	38
Qubit Ramsey coherence $T_{2,\rm q}$ (μs)	50
Qubit echo coherence $T_{2\rm E,q}$ (μs)	58
Qubit thermal population	1.15%
Readout frequency $\omega_{\rm r}/2\pi$(GHz)	8.67
Readout decay rate $\kappa_{\rm r}/2\pi$(MHz)	3.8
Readout dispersive shift $\chi_{\rm qr}/2\pi$(MHz)	2.0

nian expressed as:

$$\begin{aligned} H/\hbar \;&=\; \omega_{\rm c} a^\dagger a - \frac{K}{2} {a^\dagger}^2 a^2 \\ &+\; \omega_{\rm q} q^\dagger q - \frac{\eta_{\rm q}}{2} {q^\dagger}^2 q^2 - \chi_{\rm qc} a^\dagger a q^\dagger q \\ &+\; \omega_{\rm r} a_{\rm r}^\dagger a_{\rm r} - \chi_{\rm qr} a_{\rm r}^\dagger a_{\rm r} q^\dagger q, \end{aligned} \quad (1)$$

where a,q and $a_{\rm r}$ are the annihilation operators for the storage cavity, superconducting qubit, and readout resonator, respectively; $\omega_{\rm c}$, $\omega_{\rm q}$, and $\omega_{\rm r}$ are their respective resonance frequencies; K is the Kerr nonlinearity of the storage cavity; $\eta_{\rm q}$ is the anharmonicity of the qubit; and $\chi_{\rm qc}$ and $\chi_{\rm qr}$ are the cross-Kerr interactions between the qubit and the storage cavity and readout resonator, respectively. The measured values of these parameters are summarized and listed in Supplementary Table 1.

B. Quantum dynamics in phase space

Focusing solely on the storage cavity mode, the above Hamiltonian simplifies to a Kerr nonlinear Hamiltonian $H_K = \omega_{\rm c} a^\dagger a - \frac{K}{2} {a^\dagger}^2 a^2$ (assuming $\hbar = 1$). Under this Hamiltonian, a coherent state evolves into a slightly squeezed state and then collapses in phase space [9]. By applying an engineered off-resonant microwave drive $\Omega_{\rm d}(e^{i\omega_{\rm d}t} a + e^{-i\omega_{\rm d}t} a^\dagger)$, the coherent state undergoes a cyclic squeezing dynamics evolution in phase space without collapse. Here, $\Omega_{\rm d}$ and $\omega_{\rm d}$ denote the drive strength and frequency, respectively. In the rotating frame of the oscillator frequency $\omega_{\rm c}$, the driven Kerr Hamiltonian is expressed as:

$$H_{\rm d} = \Delta_{\rm d} a^\dagger a - \frac{K}{2} {a^\dagger}^2 a^2 + \Omega_{\rm d} \left(a + a^\dagger\right), \quad (2)$$

where $\Delta_{\rm d} = \omega_{\rm c} - \omega_{\rm d}$ is the frequency detuning between the oscillator and the drive.

To investigate the cyclic quantum dynamics behavior of the detuned driven Kerr Hamiltonian, we numerically simulate the evolution of a coherent state, which is initialized using a displacement operator $D(\beta) = e^{\beta a^\dagger - \beta^* a}$, under the Hamiltonian $H_{\rm d}$ by solving the master equation. The simulated cavity state is finally displaced back to the origin in phase space using a reverse displacement operator $D(-\beta)$. In addition to the Hamiltonian in Supplementary Equation (2), higher-order Kerr nonlinear effects with strengths on the order of a few hertz are also considered in the simulations to ensure accuracy, although they are not explicitly shown here. From the simulation, we extract the expectation values of quadrature operators $X = (a + a^\dagger)/2$ and $P = -i(a - a^\dagger)/2$ during the evolution and reconstruct the quantum state trajectory of the cavity state in phase space. As shown in Supplementary Figure 2(a), the trajectory exhibits cyclic evolution, and the average photon number $\langle a^\dagger a \rangle$, depicted in Supplementary Figure 2(b), also displays periodic oscillations. This cyclic dynamical evolution can be illustrated in Supplementary Figure 2(c), where the microwave drive acts as a global linear force on the quantum state in phase space, pulling it along the negative imaginary axis. Meanwhile, the Kerr nonlinearity in the sys-

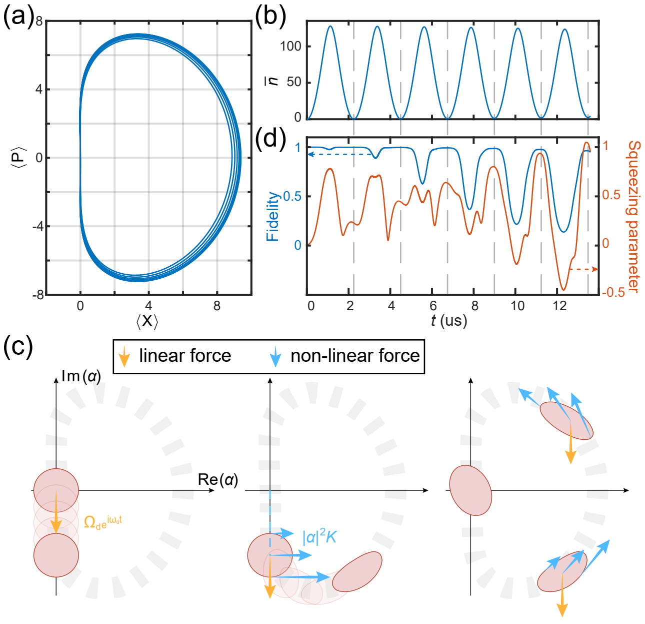

Supplementary Figure 2: Cyclic evolution of the driven Kerr nonlinear oscillator. **(a)** Quantum state trajectories for the first six evolutionary cycles in phase space. **(b)** Time evolution of the average photon number of the quantum state in the cavity. **(c)** Schematic illustration for the quantum dynamics of the detuned driven Kerr oscillator. Grey dashed lines are the evolutionary trajectories of the simulation in (a). **(d)** Time evolution of the squeezing parameters and fidelity between the evolved state and an ideal squeezed vacuum state. Dashed vertical lines correspond to the final moment of each evolution cycle.

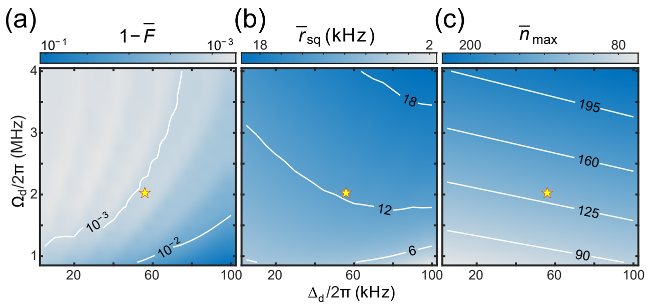

Supplementary Figure 3: Numerical simulated average infidelity of the generated squeezed states **(a)**, average squeezing rate **(b)**, and maximum average photon number **(c)** during the evolution of the cavity state as a function of the frequency detuning $\Delta_\mathrm{d} = \omega_\mathrm{c} - \omega_\mathrm{d}$ and the drive amplitude Ω_d for the first 6 cycles. The star marks the optimal choice of drive parameters for generating the squeezed Fock states in our experiment.

tem introduces an amplitude-dependent non-linear force, which rotates the state around the origin of phase space anticlockwise with an angular velocity $|\alpha|^2K$. This rotational frequency increases quadratically with the distance from the origin. As a result of these combined forces, the quantum state undergoes periodic evolution in phase space. The state trajectory maintains mirror symmetry about the real axis in phase space, which helps to cancel out undesired distortions. This process enables the transformation from a slightly curved, banana-shaped state during intermediate stages to an ideal squeezed state at the end of the evolution cycle. To verify this point, we numerically investigate both the state fidelity and squeezing parameter as a function of evolution time in Supplementary Figure 2(d). The results indicate that the fidelity reaches a maximum at the endpoint of the evolution cycle, and the squeezing parameter increases progressively with the number of cycles.

To determine the optimal driving parameters for achieving a larger squeezing level, we perform numerical simulations to investigate the performance of the evolved squeezed states after the first six evolution cycles under the Hamiltonian H_d, while scanning the frequency detuning Δ_d and the strength Ω_d. In the simulation, the cavity is initialized in a coherent state $|\beta = 2\rangle$. Here, the choice of $\beta = 2$ represents a trade-off that balances the squeezing performance and higher-order Kerr nonlinear effects. A smaller displacement amplitude β results in an insufficient squeezing rate, whereas a larger β amplifies higher-order Kerr nonlinearities, which can deform the squeezed states. We evaluate the average infidelity $1 - \bar{F}$ of the evolved squeezed states after the first six evolution cycles compared to ideal squeezed states as a function of Δ_d and Ω_d, with the results shown in Supplementary Figure 3(a). The average squeezing rate $\bar{r}_\mathrm{sq}$ is also calculated using the squeezing parameters and cyclic periods of the evolution, as shown in Supplementary Figure 3(b). Additionally, we extract the maximum average photon number $\bar{n}_\mathrm{max}$ of the cavity states during the cyclic squeezing evolution, as shown in Supplementary Figure 3(c). The simulation results indicate that within the parameter space, higher squeezing rates and fidelities are associated with larger photon numbers during the evolution, which, however, can break the dispersive approximation between the ancillary qubit and the cavity [1], similar to the case discussed in Supplementary Note 6. The optimal driving parameters used in the experiment are chosen by balancing the above three physical quantities $\bar{n}_\mathrm{max}$, r_sq and $\bar{F}$ in the simulation. As a result, we choose the drive frequency detuning of $\Delta_\mathrm{d}/2\pi = 56$kHz and drive strength of $\Omega_\mathrm{d}/2\pi = 2.01$MHz (marked by a star in Supplementary Figure 3), which yield a squeezing fidelity $\bar{F} = 99.89\%$ and an average squeezing rate $r_\mathrm{sq}/2\pi = 12.22$ kHz after the first six evolution cycles in numerical simulation.

C. Analysis of the cycling evolutionary period

1. *Semiclassical approximation*

The cyclic dynamical evolution demonstrated in Supplementary Figure 2 can be analyzed through the periodicity revealed by the Heisenberg equations of motion for X and P, that is, $\mathrm{d}X/\mathrm{d}t = -i[X, H_d]$, $\mathrm{d}P/\mathrm{d}t = -i[P, H_d]$. Based on Ehrenfest's theorem and employing a semiclassical approximation, the differential equations can be reformulated as:

$$\begin{aligned} \frac{\mathrm{d}x}{\mathrm{d}t} &= \Delta_d p - Kp(p^2 + x^2), \\ \frac{\mathrm{d}p}{\mathrm{d}t} &= -\Delta_d x + Kx(p^2 + x^2) - \Omega_d, \end{aligned} \tag{3}$$

where $x = \langle X \rangle$ and $p = \langle P \rangle$.

Through dimensionless treatment with $\epsilon = \Delta_\mathrm{d}/\Omega_\mathrm{d}$, $k = K/\Omega_\mathrm{d}$ and $\tau = t\Omega_\mathrm{d}$, Supplementary Equations (3) can be reformulated as

$$\begin{aligned} \frac{\mathrm{d}x}{\mathrm{d}\tau} &= \epsilon p - kp(p^2 + x^2), \\ \frac{\mathrm{d}p}{\mathrm{d}\tau} &= -\epsilon x + kx(p^2 + x^2) - 1. \end{aligned} \tag{4}$$

Given that $\Delta_\mathrm{d} \ll \Omega_\mathrm{d}$ such that $\epsilon \ll 1$, we assume that the solution of Supplementary Equations (4) can be expanded in a perturbative series as

$$\begin{aligned} x(\tau) &= x_0(\tau) + \epsilon x_1(\tau) + \mathcal{O}(\epsilon^2), \\ p(\tau) &= p_0(\tau) + \epsilon p_1(\tau) + \mathcal{O}(\epsilon^2), \end{aligned} \tag{5}$$

where x_0, x_1, p_0 and p_1 are all independent of ϵ. Substituting Supplementary Equations (5) into Supplementary Equations (4) and retaining the zeroth-order and first-order terms in ϵ, we obtain

$$\begin{aligned} \frac{\mathrm{d}x_0}{\mathrm{d}\tau} &= -kp_0(p_0^2 + x_0^2), \\ \frac{\mathrm{d}p_0}{\mathrm{d}\tau} &= kx_0(p_0^2 + x_0^2) - 1, \end{aligned} \tag{6}$$

and

$$\begin{aligned}\frac{\mathrm{d}x_1}{\mathrm{d}\tau} &= p_0 - 2kp_0x_0x_1 - kp_1(3p_0^2 + x_0^2),\\ \frac{\mathrm{d}p_1}{\mathrm{d}\tau} &= -x_0 + 2kx_0p_0p_1 + kx_1(p_0^2 + 3x_0^2).\end{aligned} \qquad (7)$$

Solving Supplementary Equations (6), we obtain a constant solution $x_0 = k^{-1/3}$, $p_0 = 0$. Substituting these into Supplementary Equations (7) yields a general solution as:

$$\begin{aligned}x_1(\tau) &= \mathcal{C}_1 \cos(\omega_\tau \tau) + \frac{1}{3k^{2/3}},\\ p_1(\tau) &= \mathcal{C}_2 \sin(\omega_\tau \tau),\end{aligned} \qquad (8)$$

where $\omega_\tau = \sqrt{3}k^{1/3}$, and $\mathcal{C}_1$ and $\mathcal{C}_2$ are undetermined coefficients related to the initial conditions. Consequently, the evolutionary period is given by

$$T_c = 2\pi/(\omega_\tau \Omega_d) = 2\pi/(\sqrt{3}K^{1/3}\Omega_d^{2/3}). \qquad (9)$$

2. *Numerical simulation*

In addition, we also perform systematic numerical simulations to investigate the relationship between the evolutionary period T_c and parameters K, $|\beta|^2$, Ω_d, and Δ_d. This is achieved by solving the master equation with varying these parameters across a broad parameter range. The simulated results are presented in Supplementary Figure 2.

As shown in Supplementary Figure 2(a), the coherent state amplitude β and the frequency detuning Δ_d have little impact on the period of the cyclic dynamical evolution. In contrast, the Kerr nonlinearity K and the drive strength Ω_d can significantly alter the evolution period. This relationship is illustrated in Supplementary Figure 4(b), where a linear fit on a logarithmic-logarithmic scale indicates a power-law dependence of the period T_c on K (with an exponent of approximately $-1/3$) and on Ω_d (with an exponent of approximately $-2/3$).

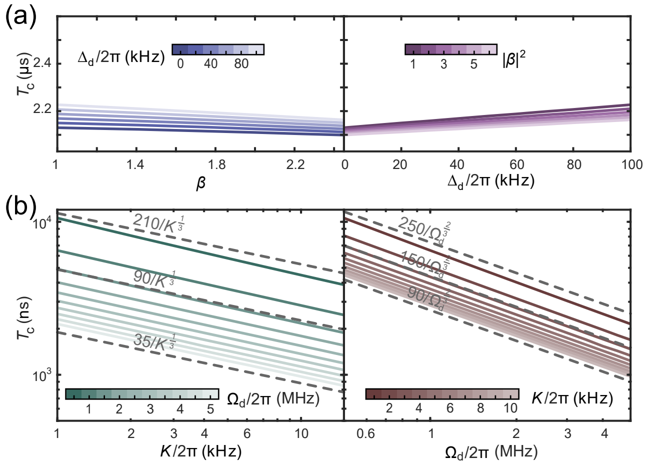

Supplementary Figure 4: Numerically simulated evolutionary period of the quantum dynamics as a function of the coherent amplitude β, frequency detuning Δ_d **(a)**, and Kerr nonlinearity K, drive strength Ω_d **(b)**. Dashed lines serve as reference for the T_c versus $K^{-1/3}$ ($\Omega_\mathrm{d}^{-2/3}$) relationship on a logarithmic scale.

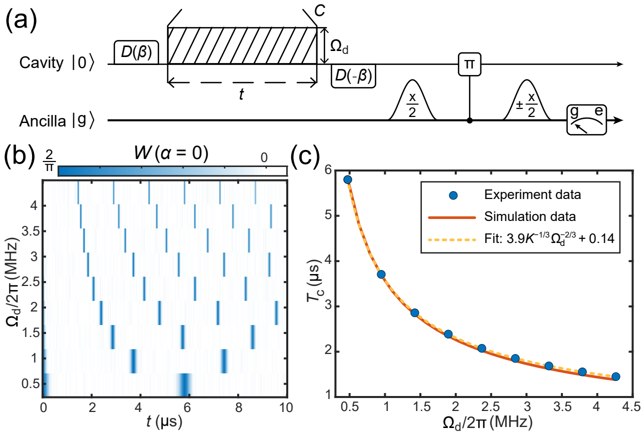

Supplementary Figure 5: Experimental characterization of the cyclic evolution of the driven Kerr nonlinear oscillator. **(a)** Experimental sequence for demonstrating the cyclic evolutionary dynamics of the detuned driven Kerr Hamiltonian. **(b)** Measured Wigner functions of the cavity state at the origin point in phase space as a function of the evolution time t and the drive amplitude Ω_d. **(c)** Quantum evolution period extracted from (b) as a function of the drive strength Ω_d, exhibiting good agreement with the simulation results (solid line). The yellow dashed line is a fitting of the expression $T_\mathrm{c} = aK^{-1/3}\Omega_\mathrm{d}^{-2/3} + b$ with fitting parameters $a = 3.9$ and $b = 0.14$.

3. *Experimental demonstration*

The cyclic evolutionary dynamics are also demonstrated experimentally, with the experimental sequence shown in Supplementary Figure 5(a). The quantum dynamics of the Wigner function at the origin point in phase space are experimentally measured as a function of the drive strength Ω_d. The experimental results shown in Supplementary Figure 5(b) indicate that the cyclic period of the quantum state evolution decreases as the drive strength Ω_d increases. We extract the evolutionary period T_c as a function of Ω_d, as shown in Supplementary Figure 5(c). The experimental results agree well with numerical simulations and fit well with the approximate relationship of $T_\mathrm{c} \propto K^{-1/3}\Omega_\mathrm{d}^{-2/3}$, indicating the successful modeling of the quantum dynamics of the detuned driven Kerr Hamiltonian.

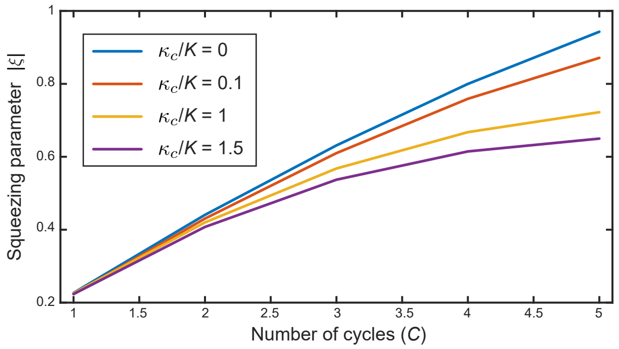

Supplementary Figure 6: Numerical simulated squeezing parameters as a function of the evolution cycles for various ratios of κ_c/K.

D. Influence of single-photon loss

Additionally, the demonstrated quantum squeezing amplification method is also applicable in scenarios where the Kerr nonlinearity K is extremely weak, even smaller than the single-photon decay rate κ_c of the storage cavity mode. To clarify this point, we conduct a numerical simulation that accounts for photon loss effects by solving the master equation:

$$\frac{\mathrm{d}\rho}{\mathrm{d}t} = -i[H_\mathrm{d}, \rho] + \kappa_c \mathcal{D}[a]\rho, \tag{10}$$

where $\mathcal{D}[a]\rho = a\rho a^\dagger - 1/2(a^\dagger a\rho + \rho a^\dagger a)$ is the Lindblad dissipative superoperator describing single-photon loss at a rate of κ_c. In the simulation, we vary the ratio of κ_c/K and calculate the squeezing parameters $|\xi|$ as a function of the number of evolution cycles while adjusting the driving parameters to ensure similar squeezing parameters after the first evolution cycle for $\beta = 2$. The simulation results, shown in Supplementary Figure 6, clearly reveal that the squeezing parameter increases with the number of cycles for different ratios of κ_c/K, demonstrating the effectiveness of the squeezing amplification scheme with an extremely weak Kerr nonlinearity.

Supplementary Note 3 - Calculating the squeezing parameters

The generated squeezed states at each evolution cycle are characterized by measuring their Wigner functions and comparing them to those of ideal squeezed states. A general squeezed Fock state can be expressed as

$$|\xi, N\rangle = S(\xi)|N\rangle, \tag{11}$$

where $S(\xi) = \exp\left(\frac{\xi^*}{2}a^2 - \frac{\xi}{2}a^{\dagger 2}\right)$ represents the squeezing operator, $\xi = |\xi|e^{i\varphi}$ is the squeezing parameter with amplitude $|\xi|$ and phase φ, and $|N\rangle$ is the multiphoton Fock state with photon number N. The Wigner function [10] of this state can be written as

$$W(\alpha) = \frac{2}{\pi}(-1)^N e^{-2|\nu|^2}\mathcal{L}_N(4|\nu|^2), \tag{12}$$

where $\nu = \cosh(|\xi|)\alpha^* + e^{-i\varphi}\sinh(|\xi|)\alpha$, and $\mathcal{L}_N$ denotes the N-th order Laguerre polynomial function. We use this function to perform a two-dimensional (2D) fit to the measured Wigner functions of the generated squeezed multiphoton Fock states. In Supplementary Figure 7(a), we present the 2D fitting results for the measured Wigner functions of the generated squeezed Fock states $|N\rangle$ (with $N = 0, 1, 2,$ and 6) after the second cycle ($C = 2$). The fitted results agree well with the experimentally measured Wigner functions. The squeezing parameters ξ of the generated squeezed Fock states are finally extracted

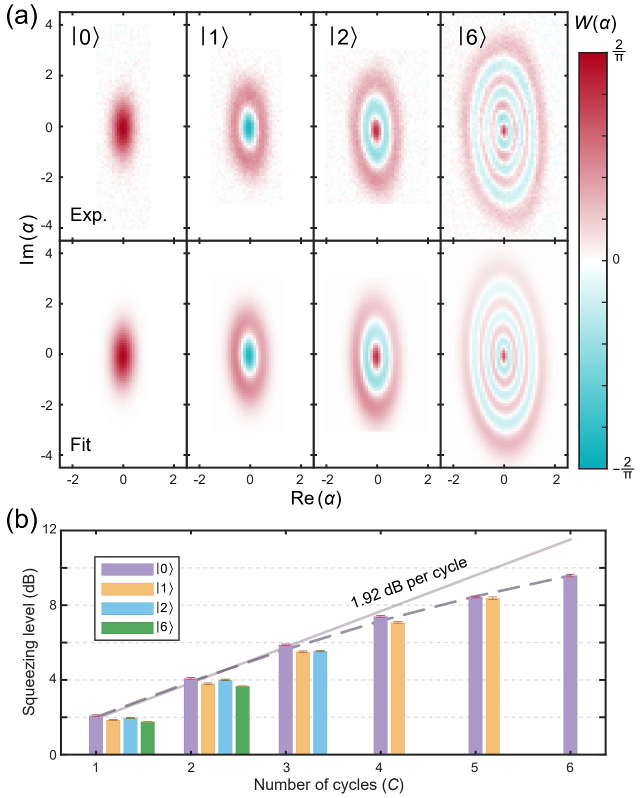

Supplementary Figure 7: Generation and characterization of squeezed Fock states with the driven squeezing approach. **(a)** The measured Wigner functions (up row) and their corresponding 2D fitting results (bottom row) for the generated squeezed Fock states $|N\rangle$ (with $N = 0, 1, 2,$ and 6) after the second cycle $C = 2$. **(b)** The extracted squeezing levels obtained from 2D fits to the measured Wigner functions of the generated squeezed Fock states as a function of the number of cycles. Error bars are the estimated 95% confidence intervals of the fittings. The solid line is a linear fit to the squeezing level for vacuum states in the linear region, showing an average addition in squeezing level of 1.92 dB per cycle. The dashed line represents squeezing levels for vacuum states from numerical simulation.

from the fitting results, as presented in Supplementary Figure 7(b).

However, this 2D Wigner function fitting method requires measuring the full Wigner function of the cavity state to ensure a reliable extraction of the squeezing parameter ξ, and thus is time-consuming in the experiment. Here, we propose to perform a global fit on the one-dimensional (1D) Wigner function cuts along two orthogonal quadratures for the generated squeezed states. In our experiment, we first eliminate the rotation phase φ of the squeezed state according to the method described in Supplementary Note 4 B, and then measure the 1D Wigner function cuts along the real axis ($\mathrm{Im}(\alpha) = 0$) and the imaginary axis ($\mathrm{Re}(\alpha) = 0$), respectively. The ideal expressions for these two 1D Wigner function cuts can be derived as $W_{\mathrm{sq}}(\alpha) = \frac{2}{\pi}\exp\left(-2e^{2|\xi|}\alpha^2\right)$ for the squeezing quadrature and $W_{\mathrm{anti-sq}}(\alpha) = \frac{2}{\pi}\exp\left(-2e^{-2|\xi|}\alpha^2\right)$ for the anti-squeezing quadrature from Supplementary Equation (12). According to these two Gaussian functions, we perform a global fit to the two measured 1D Wigner function traces to extract the squeezing parameters $|\xi|$. Supplementary Figure 8(a) presents the 1D fitting results of the squeezed states generated using the Trotterization scheme (discussed in Supplementary Note 4) with $\beta = 10$.

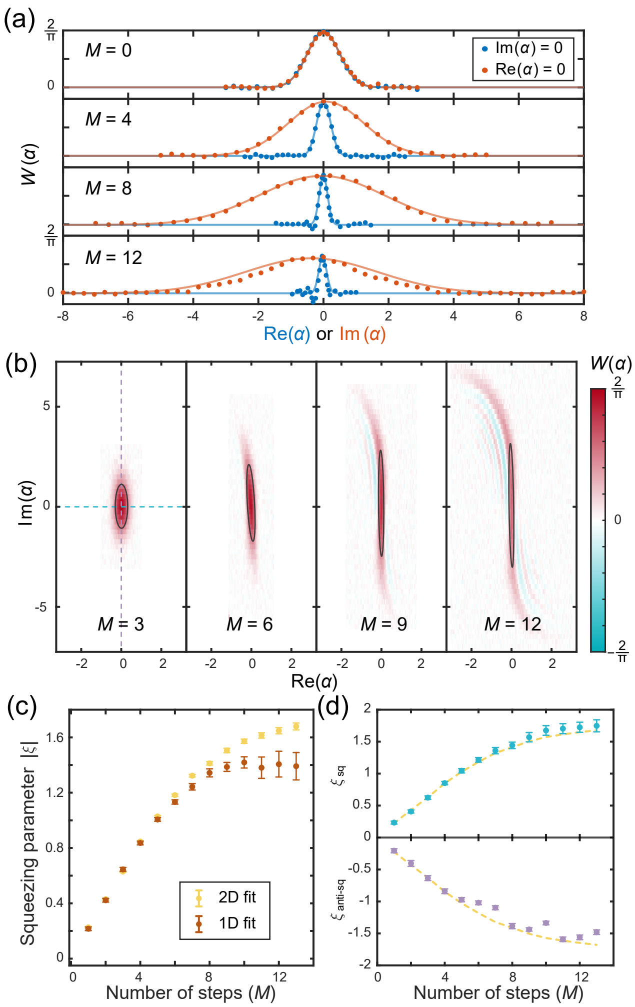

Supplementary Figure 8: Measuring squeezing parameters with both 1D and 2D Wigner function fitting methods. **(a)** Measured 1D Wigner function cuts along the real axis (blue symbols) and the imaginary axis (red symbols) for different Trotter steps with $\beta = 10$. Solid lines are global fits to the two traces. **(b)** Measured 2D Wigner functions for different Trotter steps with $\beta = 10$. The black elliptic lines represent the 2D fitting results. **(c)** Extracted squeezing parameters as a function of the number of Trotter steps with both the 1D and 2D Wigner function fitting methods shown in (a-b). **(d)** Extracted squeezing and anti-squeezing parameters from individually fitting the orthogonal quadrature cuts of the measured Wigner functions in (b). Dashed lines are the extracted squeezing parameters from 2D fit in (c). Error bars are the estimated 95% confidence intervals of the fittings.

In addition, we directly measure the full 2D Wigner functions of these generated squeezed states and perform a 2D Wigner function fit, with the fitting results indicated by black elliptical lines in Supplementary Figure 8(b). The squeezing parameters extracted from both 1D and 2D Wigner function fitting methods are compared in Supplementary Figure 8(c), yielding consistent results for small squeezing. However, for large squeezed states, the fitting results have a little discrepancy because the generated squeezed states exhibit slight deformation and rotation in phase space due to the remaining Kerr and detuning Hamiltonian terms. This leads to fitting errors in the squeezing parameters when using the 1D Wigner function fitting method. The 2D Wigner function fit accounts for residual rotation phases in the fit, thus providing more accurate fitting results for the extracted squeezing parameters.

To separately quantify the squeezing and anti-squeezing levels, we also individually fit the two orthogonal quadrature cuts of the measured Wigner function in Supplementary Figure 8(b). The individually extracted squeezing and anti-squeezing parameters as a function of Trotter steps are presented in Supplementary Figure 8(d), and are consistent with those obtained from the global 2D Wigner fits, validating the effectiveness and reliability of the fitting methodology.

Supplementary Note 4 - Quantum squeezing amplification with Trotterization technique

A. First- and second-order Trotterization

The quantum evolutionary dynamics of a coherent state $|\beta\rangle$ under the driven Kerr Hamiltonian H_{d} in Supplementary Equation (2) can be understood by performing a displacement transformation, resulting in a Hamil-

tonian:

$$\begin{aligned} H_\beta &= D(-\beta)H_\mathrm{d}D(\beta) \\ &= \Delta' a^\dagger a - \frac{K}{2}a^{\dagger 2}a^2 - \frac{K}{2}(\beta^2 a^{\dagger 2} + \beta^{*2}a^2) \\ &- K\beta a^\dagger(a^\dagger a - r) + \mathrm{H.C.}, \end{aligned} \quad (13)$$

with $\Delta' = \Delta_\mathrm{d} - 2K|\beta|^2$ and $r = \Delta_\mathrm{d}/K + \Omega_\mathrm{d}/K\beta - |\beta|^2$ representing the photon-blockade parameter. To completely eliminate the undesired photon-blockade term, we add the driven Kerr Hamiltonian in two opposite displacement frames, H_β and $H_{-\beta}$, resulting in the well-known Kerr parametric oscillator Hamiltonian:

$$\begin{aligned} H_\mathrm{KPO} &= \frac{H_\beta + H_{-\beta}}{2} \\ &= -\frac{K}{2}(\beta^2 a^{\dagger 2} + \beta^{*2}a^2) \\ &+ \Delta' a^\dagger a - \frac{K}{2}a^{\dagger 2}a^2. \end{aligned} \quad (14)$$

The time evolution of the Hamiltonian H_KPO can be approximated as

$$\begin{aligned} e^{-iH_\mathrm{KPO}T} &= (e^{-i\frac{H_{-\beta}+H_\beta}{2}\delta t})^M \\ &= (e^{-iH_{-\beta}\delta t/2}e^{-iH_\beta \delta t/2})^M \\ &+ \mathcal{O}(1/M), \end{aligned} \quad (15)$$

using the Trotterization technique. Here, $\delta t = T/M$ is the discretized evolutionary time step, and M is the number of Trotter steps. The Trotter errors in the first-order Trotter formula of Supplementary Equation (15) can be suppressed to an order of $\mathcal{O}(1/M)$ [11, 12], implying that a smaller time step would reduce the Trotter error.

In our experiment, we calibrate the Trotter time step δt by investigating the performance of quantum squeezing generation using the experimental sequence depicted in Supplementary Figure 9(a). In this sequence, the frame transformation displacement operation $D(\beta)$ has a short pulse duration of 10 ns to suppress Kerr evolution during the frame transformation. For a fixed total evolution time of 960 ns, the measured 1D Wigner function cuts along the real axis ($\mathrm{Im}(\alpha) = 0$) and the imaginary axis ($\mathrm{Re}(\alpha) = 0$) as a function of the Trotter time step δt are shown in Supplementary Figure 9(b). The experimental results clearly indicate that a smaller δt would reduce the squeezing degree, while a larger δt would increase the Trotter error. In our experiment, we choose an optimal Trotter time step of $\delta t = 80$ ns as a trade-off.

To completely eliminate the photon-blockade Hamiltonian term with the Trotterization method, the detuned drive amplitude Ω_d in H_β should be changed to $-\Omega_\mathrm{d}$ in $H_{-\beta}$ for each Trotter step. In our experiment, we set $\Omega_\mathrm{d} = 0$ to limit the average photon number of intermediate states during the evolution and engineer a virtual phase shift ϕ_d after each evolution cycle to partially counteract the accumulated phase induced by the remaining Kerr term. The optimal virtual phase shifts ϕ_d

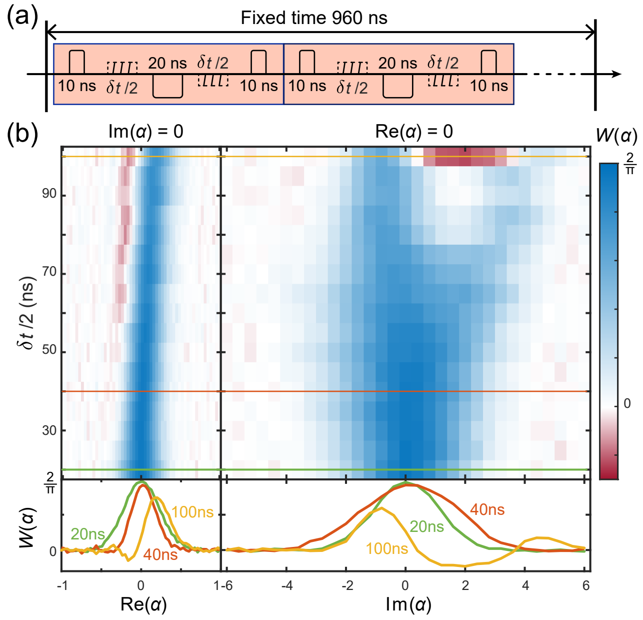

Supplementary Figure 9: Calibrating the Trotter time step δt. **(a)** Experimental sequence with a fixed total evolution time of 960 ns. **(b)** Measured 1D Wigner function cuts along the real and imaginary axes for various Trotter time steps δt with $\beta = 8$. Three horizontal cuts are provided in the bottom row, with $\delta t/2 = 40$ ns (marked by red lines) being used in our experiment in the main text.

Supplementary Table 2: Optimal virtual phase shift ϕ_d for various $|\beta|^2$ with the Trotterization method.

$\lvert\beta\rvert^2$	ϕ_d (rad)
1	$4.8\times10^{-4}\pi$
4	$4.0\times10^{-3}\pi$
16	$1.6\times10^{-2}\pi$
36	$2.8\times10^{-2}\pi$
64	$4.5\times10^{-2}\pi$
100	$5.7\times10^{-2}\pi$

for achieving the maximum squeezing parameter for various displacement amplitudes $|\beta|^2$ are determined from numerical simulations and are provided in Supplementary Table 2.

In addition, Trotter errors can be further reduced by utilizing the second-order Trotter formula:

$$\begin{aligned} e^{-iH_\mathrm{KPO}T} &= (e^{-i\frac{H_{-\beta}+H_\beta}{2}\delta t}e^{-i\frac{H_{-\beta}+H_\beta}{2}\delta t})^{\frac{M}{2}} \\ &= (e^{-iH_\beta\delta t/2}e^{-iH_{-\beta}\delta t/2}e^{-iH_{-\beta}\delta t/2}e^{-iH_\beta\delta t/2})^{\frac{M}{2}} \\ &+ \mathcal{O}(1/M^2) \end{aligned} \quad (16)$$

where Trotter errors are suppressed to the order of

$\mathcal{O}(1/M^2)$ through rearrangement sequence of H_β and $H_{-\beta}$, as illustrated in Supplementary Figure 10(a). We directly perform an experimental comparison of the first- and second-order Trotter decomposition schemes and measure the squeezing parameters of generated squeezed vacuum states. The experimental results, shown in Supplementary Figure 10(b), indicate almost identical performance for both the first- and second-order Trotter schemes. Therefore, it is unnecessary to adopt a higher-order Trotter scheme [13] in our experiment, and all experimental results presented in the main text are obtained using the first-order Trotter scheme.

B. Squeezing phase calibration

During the Kerr evolution for generating squeezed state, a rotation phase φ will accumulate due to the remaining Kerr and detuning Hamiltonian terms, resulting in a phase-space inclination of the squeezed states, as illustrated in Supplementary Figure 11(a). It is crucial to characterize and eliminate this phase to obtain an accurate squeezing parameter from the 1D Wigner function fit discussed in Supplementary Note 3. In our experiment, this phase is calibrated by measuring the Wigner function $W(\alpha)$ of the generated squeezed state as a function of the rotation phase $\arg(\alpha)$ with a fixed amplitude $|\alpha| = 0.7$. The experimental results are shown in Supplementary Figure 11(b) for the generated squeezed states at each Trotter step with $\beta = 10$. Subsequently, the rotation phase of the squeezed state at each Trotter step is extracted by identifying the rotation angle that maximizes the Wigner functions. Finally, the calibrated phases of the squeezed states are eliminated by performing virtual phase rotations in the experimental sequence. These virtual operations are implemented by adding a phase offset to the subsequent microwave pulses in the sequence and have a duration of zero [14].

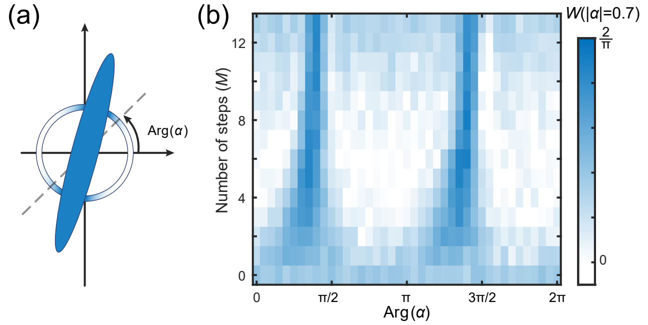

Supplementary Figure 11: Calibrating the rotation phases of generated squeezed states. **(a)** Schematic illustration for calibrating the rotation phases of generated squeezed states. The ring structure indicates the circumferential 1D Wigner functions. **(b)** Experimentally measured 1D Wigner functions $W(\alpha)$ as a function of the rotation phase $\arg(\alpha)$ with a fixed amplitude $|\alpha| = 0.7$ for various Trotter steps M with $\beta = 10$.

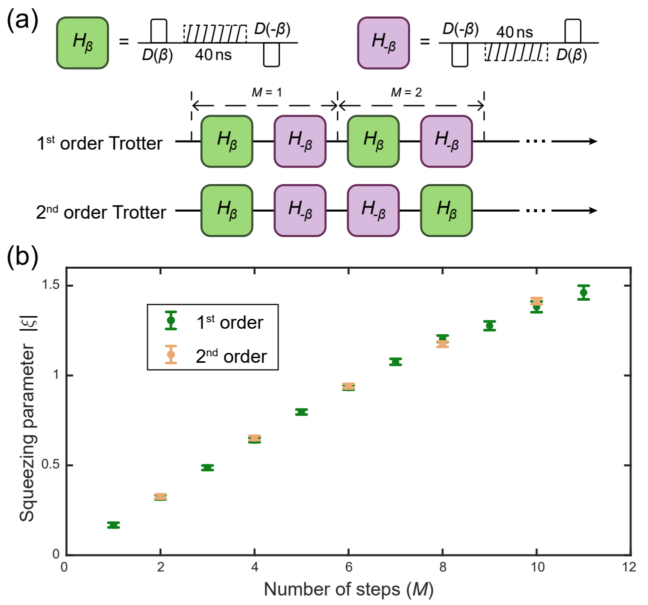

Supplementary Figure 10: Comparison of the first- and the second-order Trotter schemes. Experimental sequences **(a)** and the measurement results **(b)** for both the first- and the second-order Trotter schemes with $\beta = 8$. Error bars are the estimated 95% confidence intervals of the 1D fittings.

Supplementary Note 5 - Quantum metrology for sensing displacement

A. Parity measurement

A variety of protocols for quantum-enhanced precision measurement of small displacements have been well established, such as homodyne detection [15], projective measurement [16, 17], and parity measurement [3]. In this work, we employ the parity measurement for high-precision displacement estimation to evaluate the metrological capability of the generated squeezed states. This is achieved by first encoding a displacement amplitude α onto the generated squeezed state via a displacement operation, and then probing it through an ancilla-assisted parity measurement, as shown in Fig. 4a of the main text. The displacement amplitude can be inferred from the qubit ground state population, which relates to the Wigner function of an ideal squeezed state through the formula $P_g^{\mathrm{ideal}}(\alpha) = \frac{1}{2}[\frac{\pi}{2}W_{\mathrm{sq}}(\alpha) + 1] = \frac{1}{2}[\exp\left(-2e^{2|\xi|}\alpha^2\right) + 1]$. We fit the measured results with the probability model $P_g(\alpha) = A\exp(-2e^{2|B|}\alpha^2) + D$ to obtain a smooth probability distribution $P_g(\alpha)$, where the free parameters A and D account for experimental imperfections, and B is the extracted squeezing parameter. The corresponding classical Fisher information (FI)

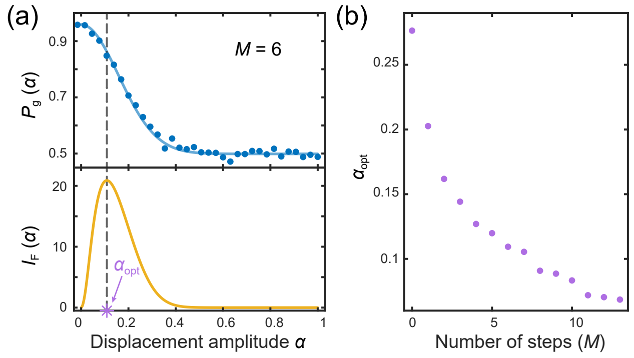

Supplementary Figure 12: Extracting the optimal displacement amplitude for quantum sensing using the Trotterization-generated squeezed states. **(a)** Measured qubit ground state populations (blue symbols), corresponding fit (blue line), and extracted FI (yellow line) as a function of the displacement amplitude α with $M = 6$. The optimal displacement amplitude $\alpha_{\rm opt}$ with maximum FI is indicated with a snowflake symbol. **(b)** Extracted optimal displacements as a function of the number of Trotter steps M.

for this measurement strategy can thus be calculated by $I_{\rm F}(\alpha) = \frac{1}{P_g(1-P_g)}(\frac{{\rm d}P_g}{{\rm d}\alpha})^2$ [18], as shown in Supplementary Figure 12(a). This metrology scheme exhibits an optimal operating point where the sensitivity to small displacement is maximized, that is $\alpha_{\rm opt} = \arg\max_\alpha I_{\rm F}(\alpha)$, as presented in Supplementary Figure 12(b). All experimental data presented in Fig. 4 of the main text and Supplementary Figure 13 are obtained at this optimal displacement amplitude.

B. Bayesian inference

Bayesian inference is a versatile and powerful methodology for parameter estimation based on probability distributions. In this work, we employ Bayesian estimation to extract the encoded small displacement amplitude α from the measured qubit ground population.

To estimate a displacement parameter α given its prior probability $P(\alpha)$, the core of the Bayesian inference lies in maximizing the posterior probability

$$P(\alpha|\mathcal{M}) \propto P(\alpha) \times P(\mathcal{M}|\alpha), \tag{17}$$

where $P(\mathcal{M}|\alpha)$ represents the likelihood of observing the ancilla in ground state ($\mathcal{M} = g$) or excited state ($\mathcal{M} = e$) under a small displacement α. Assuming a uniform prior probability $P(\alpha)$ over the displacement amplitude interval $\alpha \in [0, 0.5]$, Bayesian estimation reduces to maximum likelihood estimation (MLE). In this case, we have $P(\alpha|\mathcal{M}) \propto P(\mathcal{M}|\alpha)$. In a series of $N_{\rm meas}$ independent experiments, the ancillary qubit is measured in the ground (excited) state in N_g (N_e) instances. The corresponding likelihood function is given by $P(\mathcal{M}|\alpha)^{N_{meas}} = [P_g(\alpha)]^{N_g} \times [1 - P_g(\alpha)]^{N_e}$. In our experiment, we employ the log-likelihood function,

$$\begin{aligned} L(\mathcal{M}_1, \ldots \mathcal{M}_{N_{\rm meas}}|\alpha) &= P_g^{\rm exp} \ln(P_g(\alpha)) \\ &+ P_e^{\rm exp} \ln(1 - P_g(\alpha)), \end{aligned} \tag{18}$$

where $P_g^{\rm exp} = N_g/N_{\rm meas}$ and $P_e^{\rm exp} = N_e/N_{\rm meas}$ are the corresponding outcome probabilities. And the displacement parameter is finally estimated by maximizing the log-likelihood function through $\alpha_{\rm est} = \arg\max_\alpha L(\mathcal{M}_1, \ldots \mathcal{M}_{N_{\rm meas}}|\alpha)$. The Bayesian posterior distribution $P(\alpha|\mathcal{M})$ directly allows extracting the variance of the estimated displacement, with the extracted standard deviation shown in Supplementary Figure 13. To better quantify the correlated noise in the system, we utilize a well-established sensitive metric—Allan deviation [19]—to identify and quantify the low-frequency fluctuations, with the experimental results shown in Fig. 4b in the main text.

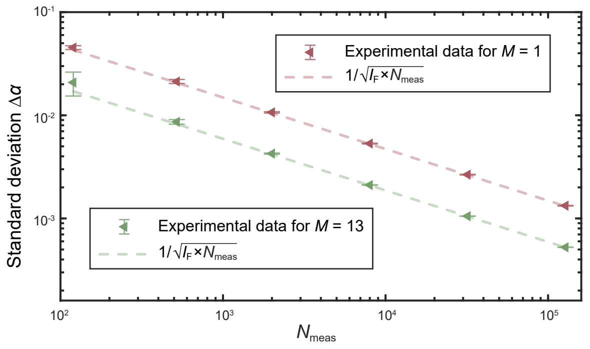

Supplementary Figure 13: Extracted standard deviation (triangles) from the Bayesian posterior distributions for estimating the displacement amplitude using squeezed states generated with different Trotter steps (red: $M = 1$, green: $M = 13$) versus the number of independent measurements $N_{\rm meas}$. Dashed lines represent the theoretical Cramér-Rao bound using classical FI. Error bars are standard deviations from repeated experiments.

Supplementary Note 6 - Breakdown of the dispersive approximation

In pursuit of a high squeezing rate with the Trotterization technique, a substantial displacement amplitude is required during the displacement frame transformation. However, this leads to excessive photon numbers in the storage cavity, potentially disrupting the dispersive approximation shown in Supplementary Equation (1). Consequently, the Jaynes-Cummings (JC) interaction between the storage cavity and the ancilla qubit should be considered, as described by the Hamiltonian:

$$\begin{aligned} H_{\rm JC} &= \omega_{\rm c}' a^\dagger a + \omega_{\rm q}' q^\dagger q - \frac{\eta_{\rm q}}{2} {q^\dagger}^2 q^2 \\ &+ g_{\rm qc}(a^\dagger q + a q^\dagger), \end{aligned} \tag{19}$$

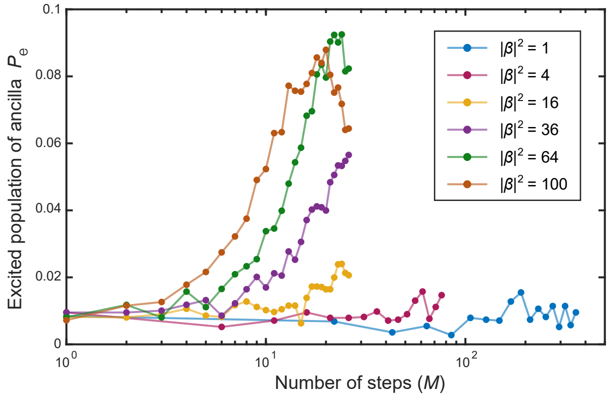

Supplementary Figure 14: Excited population of the auxiliary qubit as a function of the number of Trotter steps for various displacement amplitudes $|\beta|^2$.

where ω_c' and ω_q' are their respective bare resonance frequencies and g_{qc} is the coupling constant between the qubit and the storage cavity. This interaction can induce qubit excitations when the storage cavity contains a large number of photons. This phenomenon is also verified in our experiment by measuring the qubit excited state populations as a number of Trotter steps for various displacement amplitudes $|\beta|^2$, with the results depicted in Supplementary Figure 14. As the displacement amplitude increases, the qubit excited state population progressively increases with the number of Trotter steps. The breakdown of the dispersive approximation imposes a constraint on the maximum achievable value of β in our experiment. In addition, the qubit excited population induced by the breakdown of the dispersive approximation can diminish the state fidelity of the generated squeezed state because the entanglement between the qubit and the storage cavity during the evolution would reduce the purity of the quantum state in the storage cavity.

Supplementary Note 7 - Error analysis in the Trotter scheme

The primary error sources for the displacement-enhanced squeezing generation with the Trotterization technique include decoherence errors of the storage cavity, Trotter errors, deformation errors caused by the Kerr nonlinear term, ancillary qubit excitation errors resulting from the breakdown of the dispersive approximation, and errors due to higher-order Kerr nonlinearity. In order to estimate the contributions of these errors, we conduct numerical simulations by solving the master equation while accounting for these error sources. Supplementary Figure 15 presents both the simulated and measured state fidelities of the generated squeezed states as a function of Trotter steps with $\beta = 10$. The fidelities are determined by comparing the simulated or reconstructed density matrices to the ideal squeezed state. The Trotterized squeezing approach can benefit from the echoed displacements, which effectively suppress cavity dephasing errors during the evolution. This is confirmed by comparing the simulation results with and without including the cavity pure dephasing rate in the simulation, as presented in Supplementary Figure 15. When we set an effective cavity decoherence lifetime $T_{2,\mathrm{eff}} = 2T_{1,\mathrm{c}}$ (indicating no pure dephasing rate) in the master equation solver, the simulated fidelities are in good agreement with the measurements. This alignment confirms the effectiveness of error modeling and the suppression of cavity dephasing in our Trotterized squeezing generation method. Additionally, we find that the deformation errors induced by Kerr nonlinearity are the predominant errors, which increase with the number of Trotter steps.

Besides, we also provide a comparative analysis of simulated versus experimentally measured squeezing parameters in the inset of Supplementary Figure 15 for the generated squeezed states across various Trotter steps. When considering only the first-order $(\frac{K}{2}a^{\dagger 2}a^2)$ and second-order $(\frac{K_2}{6}a^{\dagger 3}a^3)$ Kerr nonlinearities in the simulation, we find that the simulation curve of the squeezing parameters increasingly diverges from the experimental results as the Trotter steps increase. This divergence may be attributed to errors induced by higher-order Kerr nonlinearity, such as the third-order Kerr nonlinearity term $(\frac{K_3}{24}a^{\dagger 4}a^4)$. Here, K_2 and K_3 denote the strengths of the second-order and third-order Kerr effects, respectively. Given that this extremely weak higher-order Kerr nonlinearity is beyond our experimental characterization preci-

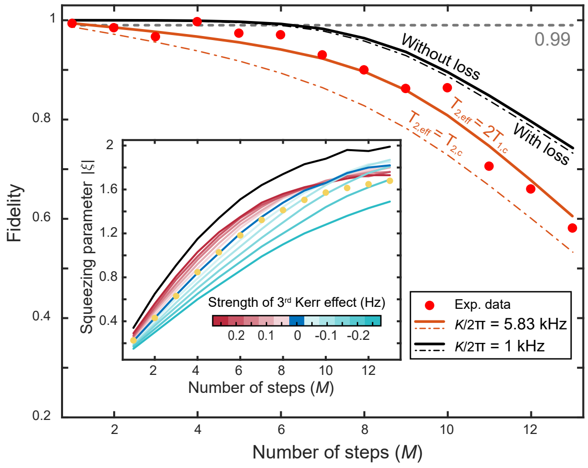

Supplementary Figure 15: Error analysis of the generated squeezed states with Trotterization technique. Measured (red circles) and simulated (solid and dashed lines) state fidelities between the generated quantum states and ideal squeezed states as a function of the Trotter steps. The inset presents the squeezing parameters (yellow circles) extracted from 2D Wigner function fits shown in Supplementary Figure 8(c). Solid lines in the inset show the simulation results with varying the third-order Kerr nonlinearity in the simulation. Black lines represent the simulations with a Kerr nonlinearity of $K/2\pi = 1$ kHz.

sion, we investigate its influence by performing numerical simulations with varying the strengths of the third-order nonlinearity. The simulation results shown in the inset capture the behavior of the measured squeezing parameters over the Trotter steps.

Supplementary Note 8 - Squeezing approaches comparison

In this section, we analyze and compare various methods for generating microwave squeezed states in superconducting quantum circuits. In this system, flying squeezed microwave states have been successfully generated using Josephson parametric amplifiers, achieving a maximum squeezing of 11.4 dB [20, 21]. Besides, microwave states within the cavity in this system can also be compressed to realize two-photon squeezing operations. This can be achieved by employing a superconducting qubit through measurement and post-selection, resulting in a squeezing of 5.7 dB [22]. These intracavity squeezed states can also be generated using a parametric mixing pump of the nonlinear Josephson elements [23–25], achieving squeezed microwave phononic states beyond 3 dB [24] and photonic states up to 8.63 dB squeezing [25]. Furthermore, parameterized quantum circuits with echoed conditional displacements have been employed to generate squeezed states within the microwave cavity, reaching a squeezing up to 11.1 dB [26, 27].

In comparison to these squeezing methods, our Trotterized squeezed approach generates squeezed states deterministically, utilizing simple and natively available gates and dynamics. These generated squeezed states within the cavity differ from itinerant squeezing due to the long coherence lifetime of the cavity mode, enabling efficient quantum information processing with these intracavity squeezing resources. Unlike other squeezing approaches, our method eliminates decoherence errors induced by the ancilla qubit during the evolution. Additionally, the echoed displacement operations effectively suppress cavity dephasing errors during the evolution. With these advantages, our squeezing approach achieves a maximum squeezing degree of 14.6 dB, which, to our knowledge, is the largest squeezing value for intracavity microwave photonic states.

[1] A. Blais, A. L. Grimsmo, S. M. Girvin, and A. Wallraff, "Circuit quantum electrodynamics," Rev. Mod. Phys. **93**, 025005 (2021).

[2] Z. Ni, S. Li, X. Deng, Y. Cai, L. Zhang, W. Wang, Z.-B. Yang, H. Yu, F. Yan, S. Liu, C.-L. Zou, L. Sun, S.-B. Zheng, Y. Xu, and D. Yu, "Beating the break-even point with a discrete-variable-encoded logical qubit," Nature **616**, 56 (2023).

[3] X. Deng, S. Li, Z.-J. Chen, Z. Ni, Y. Cai, J. Mai, L. Zhang, P. Zheng, H. Yu, C.-L. Zou, S. Liu, F. Yan, Y. Xu, and D. Yu, "Quantum-enhanced metrology with large Fock states," Nat. Phys. **20**, 1874 (2024).

[4] M. Reagor, W. Pfaff, C. Axline, R. W. Heeres, N. Ofek, K. Sliwa, E. Holland, C. Wang, J. Blumoff, K. Chou, M. J. Hatridge, L. Frunzio, M. H. Devoret, L. Jiang, and R. J. Schoelkopf, "Quantum memory with millisecond coherence in circuit QED," Phys. Rev. B **94**, 014506 (2016).

[5] J. Koch, T. M. Yu, J. Gambetta, A. A. Houck, D. I. Schuster, J. Majer, A. Blais, M. H. Devoret, S. M. Girvin, and R. J. Schoelkopf, "Charge-insensitive qubit design derived from the Cooper pair box," Phys. Rev. A **76**, 042319 (2007).

[6] C. Axline, M. Reagor, R. Heeres, P. Reinhold, C. Wang, K. Shain, W. Pfaff, Y. Chu, L. Frunzio, and R. J. Schoelkopf, "An architecture for integrating planar and 3D cQED devices," Appl. Phys. Lett. **109**, 042601 (2016).

[7] M. Reagor, H. Paik, G. Catelani, L. Sun, C. Axline, E. Holland, I. M. Pop, N. A. Masluk, T. Brecht, L. Frunzio, M. H. Devoret, L. Glazman, and R. J. Schoelkopf, "Reaching 10 ms single photon lifetimes for superconducting aluminum cavities," Appl. Phys. Lett. **102**, 192604 (2013).

[8] Z. Ni, S. Li, L. Zhang, J. Chu, J. Niu, T. Yan, X. Deng, L. Hu, J. Li, Y. Zhong, S. Liu, F. Yan, Y. Xu, and D. Yu, "Scalable Method for Eliminating Residual ZZ Interaction between Superconducting Qubits," Phys. Rev. Lett. **129**, 040502 (2022).

[9] G. Kirchmair, B. Vlastakis, Z. Leghtas, S. E. Nigg, H. Paik, E. Ginossar, M. Mirrahimi, L. Frunzio, S. M. Girvin, and R. J. Schoelkopf, "Observation of quantum state collapse and revival due to the single-photon Kerr effect," Nature **495**, 205 (2013).

[10] G. M. A. Al-Kader, "Phase distribution from the Wigner function for superpositions of squeezed displaced Fock states," J. Opt. B: Quantum Semiclass. Opt. **5**, S228 (2003).

[11] M. Suzuki, "Generalized Trotter's formula and systematic approximants of exponential operators and inner derivations with applications to many-body problems," Commun. Math. Phys. **51**, 183 (1976).

[12] J. Han, W. Cai, L. Hu, X. Mu, Y. Ma, Y. Xu, W. Wang, H. Wang, Y. P. Song, C.-L. Zou, and L. Sun, "Experimental simulation of open quantum system dynamics via trotterization," Phys. Rev. Lett. **127**, 020504 (2021).

[13] M. Suzuki, "Improved Trotter-like formula," Phys. Lett. A **180**, 232 (1993).

[14] D. C. McKay, C. J. Wood, S. Sheldon, J. M. Chow, and J. M. Gambetta, "Efficient Z gates for quantum computing," Phys. Rev. A **96**, 022330 (2017).

[15] M. Fadel, N. Roux, and M. Gessner, "Quantum metrology with a continuous-variable system," arXiv: 2411.04122 (2024).

[16] X. Pan, T. Krisnanda, A. Duina, K. Park, P. Song, C. Y. Fontaine, A. Copetudo, R. Filip, and Y. Y. Gao, "Realization of Versatile and Effective Quantum Metrology Using a Single Bosonic Mode," PRX Quantum **6**, 010304 (2025).

[17] K. Park, T. Krisnanda, Y. Gao, and R. Filip, "Quantum phase estimation beyond the gaussian limit," arXiv:

2508.13046 (2025).

[18] M. G. A. PARIS, "Quantum estimation for quantum technology," Int. J. Quantum Inf. **07**, 125 (2009).

[19] D. W. Allan, "Should the classical variance be used as a basic measure in standards metrology?" IEEE Trans. Instrum. Meas. **IM-36**, 646 (1987).

[20] B. Yurke, P. G. Kaminsky, R. E. Miller, E. A. Whittaker, A. D. Smith, A. H. Silver, and R. W. Simon, "Observation of 4.2-K equilibrium-noise squeezing via a Josephson-parametric amplifier," Phys. Rev. Lett. **60**, 764 (1988).

[21] J. Y. Qiu, A. Grimsmo, K. Peng, B. Kannan, B. Lienhard, Y. Sung, P. Krantz, V. Bolkhovsky, G. Calusine, D. Kim, A. Melville, B. M. Niedzielski, J. Yoder, M. E. Schwartz, T. P. Orlando, I. Siddiqi, S. Gustavsson, K. P. O'Brien, and W. D. Oliver, "Broadband squeezed microwaves and amplification with a Josephson travelling-wave parametric amplifier," Nat. Phys. **19**, 706 (2023).

[22] W. Wang, L. Hu, Y. Xu, K. Liu, Y. Ma, S.-B. Zheng, R. Vijay, Y. P. Song, L.-M. Duan, and L. Sun, "Converting Quasiclassical States into Arbitrary Fock State Superpositions in a Superconducting Circuit," Phys. Rev. Lett. **118**, 223604 (2017).

[23] R. Dassonneville, R. Assouly, T. Peronnin, A. Clerk, A. Bienfait, and B. Huard, "Dissipative Stabilization of Squeezing Beyond 3 dB in a Microwave Mode," PRX Quantum **2**, 020323 (2021).

[24] S. Marti, U. von Lüpke, O. Joshi, Y. Yang, M. Bild, A. Omahen, Y. Chu, and M. Fadel, "Quantum squeezing in a nonlinear mechanical oscillator," Nat. Phys. **20**, 1448 (2024).

[25] A. M. Eriksson, T. Sépulcre, M. Kervinen, T. Hillmann, M. Kudra, S. Dupouy, Y. Lu, M. Khanahmadi, J. Yang, C. Castillo-Moreno, P. Delsing, and S. Gasparinetti, "Universal control of a bosonic mode via drive-activated native cubic interactions," Nat. Commun. **15**, 2512 (2024).

[26] A. Eickbusch, V. Sivak, A. Z. Ding, S. S. Elder, S. R. Jha, J. Venkatraman, B. Royer, S. M. Girvin, R. J. Schoelkopf, and M. H. Devoret, "Fast universal control of an oscillator with weak dispersive coupling to a qubit," Nat. Phys. **18**, 1464 (2022).

[27] X. Pan, J. Schwinger, N.-N. Huang, P. Song, W. Chua, F. Hanamura, A. Joshi, F. Valadares, R. Filip, and Y. Y. Gao, "Protecting the Quantum Interference of Cat States by Phase-Space Compression," Phys. Rev. X **13**, 021004 (2023).
